# Intra-population differences of apolipoproteins in the aqueous humor

**DOI:** 10.1186/s12944-021-01555-0

**Published:** 2021-10-03

**Authors:** Parth A. Patel, Tae Jin Lee, Sai Karthik Kodeboyina, Garrett Jones, Kathryn Bollinger, Lane Ulrich, David Bogorad, Amy Estes, Wenbo Zhi, Shruti Sharma, Ashok Sharma

**Affiliations:** 1 Center for Biotechnology and Genomic Medicine, Medical College of Georgia, Augusta University, 1120 15th Street, CA4094, Augusta, GA 30912 USA; 2 Mass General Brigham, 215 First Street, Cambridge, MA 02142 USA; 3 Department of Ophthalmology, Medical College of Georgia, Augusta University, Augusta, GA 30912 USA; 4 Department of Population Health Sciences, Medical College of Georgia, Augusta University, Augusta, GA 30912 USA

## Abstract

**Background:**

Evidence suggests that proteins related to lipid metabolism, such as apolipoproteins, play an important role in the maintenance of normal vision. While several members of the apolipoprotein family are abundant in human aqueous humor (AH), their study remains difficult due to the AH’s small volume, low protein concentration, and the invasive nature of sample collection. In this study, we report the use of Liquid Chromatography Tandem Mass Spectrometry (LC-MS/MS) to discover associations between AH apolipoproteins and race, gender, and ocular structure in patients with and without primary open angle glaucoma (POAG).

**Methods:**

AH samples were collected from 231 patients undergoing phacoemulsification or glaucoma incisional surgery at the Medical College of Georgia, Augusta University and subsequently analyzed via LC-MS/MS. The number of peptide spectrum matches (PSMs) for each protein was used as a semi-quantitative measure of relative protein levels. Parameters related to ocular structure were determined using Optical Coherence Tomography (OCT) and Heidelberg Retinal Tomography (HRT). These data sets were probed for relationships between apolipoprotein levels and POAG, demographics (gender and race), and ocular structure.

**Results:**

A total of ten apolipoproteins were detected in the 231 collected AH samples, with six detected in 100% of the samples, one detected in almost 57% of the samples and three detected in less than 10% of the samples. The levels of APOA1, APOC3, and APOD were higher among POAG subjects. Stratification by gender and race revealed demographic-specific variations. The levels of five apolipoproteins (APOA1, APOA2, APOA4, APOC3, and APOD) were higher in female POAG patients, whereas no apolipoprotein levels were altered in male POAG patients. The levels of APOA1, APOA2, APOA4, and APOD were increased in glaucomatous African American patients, whereas APOE and APOH levels were decreased in glaucomatous Caucasian patients. We also found distinct associations between apolipoprotein levels and OCT and HRT parameters in patients with and without POAG.

**Conclusions:**

The intra-population variation in apolipoprotein levels highlights the heterogeneity of glaucoma as a disease, suggesting the importance of personalized treatments. Gender and race-specific alterations may be associated with higher risks of POAG in females and members of the African American population.

**Supplementary Information:**

The online version contains supplementary material available at 10.1186/s12944-021-01555-0.

## Introduction

Aqueous humor (AH) is the clear fluid located within the anterior and posterior chambers of the eye. The key functions of AH include maintenance of refraction, ocular shape, intraocular pressure, oxygen management, nutrient delivery, and metabolic waste removal [1]. To further establish an understanding of these homeostatic processes, research efforts have sought to delineate the AH proteome [1–4]. Apolipoproteins have been identified as a prominent category in this proteome.

Apolipoproteins carry out many intraocular functions. By binding to lipids, they enable transport of those lipids through various hydrophilic biological domains. When acting as enzymatic cofactors, they are involved in the metabolism of lipoproteins [5, 6]. Through structural and functional complexity, these entities are classified into numerous classes and subclasses, each with a unique role in the complex biological milieu of the human body.

Several studies have examined the relationship between apolipoproteins and ocular conditions including glaucoma, cataracts, pseudo-exfoliation syndrome, and age-related macular degeneration [7–12]. APOA1, APOC3, and APOE have been shown to be elevated in the AH of POAG patients as compared to patients with cataracts [10, 12]. Previous studies have also reported substantial gender-specific differences in plasma apolipoprotein concentrations [13, 14]. However, prior research exploring gender and race variations in the AH proteome has been limited by relatively small sample sizes [15].

In the present study, we quantified differences in levels of apolipoproteins in 231 subjects based on disease state (POAG vs. non-glaucomatous cataract), gender, race, and ocular structure, as assessed by Heidelberg Retinal Tomography (HRT) and optical coherence tomography (OCT). We found gender and race-specific associations between AH apolipoproteins and POAG. Furthermore, the correlations of apolipoproteins with ocular structure were distinct among the POAG and cataract subgroups.

## Materials and methods

### Subjects

AH samples were obtained from 231 subjects undergoing ocular surgery at the Medical College of Georgia, Augusta University. Samples were collected by aspiration of AH from the anterior chamber after the initial clear corneal incision was made during cataract and glaucoma surgeries. This collection does not involve any additional ocular procedures or any alteration in the amount or method of AH removal. The AH samples were placed in sealed sterile tubes, then frozen at − 80 °C until processing. The Institutional Review Board of Augusta University has approved the study (IRB #611480) and written, informed consents were obtained from all study participants. In total, 158 AH samples from cataract surgery patients and 73 AH samples from POAG patients were collected. No significant differences in race, gender, or IOP levels were observed between these groups (Table 1).

**Table 1:** Demographic information of subjects

Demographic Characteristic	Cataract (*n* = 158)	POAG (*n* = 73)	*p*-value
Age (years, mean ± SD)	67.65 ± 8.91	68.79 ± 10.79	0.43^a^
Gender (F/M)	100/58	43/30	0.62^b^
Race (African Americans/Caucasians)	95/63	44/29	1^b^

^a^two-sample t-test

^b^chi-square test

### LC-MS/MS analysis

AH samples (60 μL) were successively lyophilized and reconstituted in 30 μL of 8 M urea in 50 mM Tris-HCl (pH 8). Cysteine residues were reduced with 20 mM dithiothreitol, alkylated with 55 mM iodoacetemide, followed by the addition of 240 μL of 50 mM ammonium bicarbonate buffer to decrease the urea concentration to less than 1 mM. Protein concentration was measured using a Bradford assay kit (Pierce, Rockford, IL), according to the manufacturer’s instructions. Finally, protein digestion was performed by the addition of a 1:20 ratio (w/w) of trypsin and maintained overnight at 37 C°.

The digested AH samples were cleaned on a C18 spin plate (Nest Group, Southborough, MA, USA), separated via an Ultimate 3000 nano-UPLC system (Thermo Scientific), and analyzed with an Orbitrap Fusion Tribrid mass spectrometer (Thermo Scientific). The reconstituted peptide mixture (6 μL) was trapped and washed on a Pepmap100 C18 trap (5 μm, 0.3 × 5 mm) for 10 min at a rate of 20 μL/min using 2% acetonitrile in water with 0.1% formic acid. The peptides were then separated on a Pepman100 RSLC C18 column (2.0 μm, 75 μm × 150 mm) using a gradient of 2 to 40% acetonitrile with 0.1% formic acid over 120 min (flow rate: 300 nL/min; column temperature: 40֯ C). Eluted peptides were analyzed via data-dependent acquisition in positive mode using the following settings: Orbitrap MS analyzer for precursor scan at 120,000 FWHM from 300 to 1500 m/z; Ion-trap MS analyzer for MS/MS scans in top speed mode (2-s cycle time) with dynamic exclusion settings (repeat count: 1; repeat duration: 15 s; exclusion duration: 30 s). The fragmentation method was collision-induced dissociation (CID) with a normalized collision energy of 30%.

### Protein identification and quantification

For the purposes of protein identification and quantification, raw MS data were processed via the Proteome Discoverer (v1.4; Thermo Scientific) and searched using the SequestHT algorithm against the Uniprot-SwissProt human database with 20,385 entries. Search parameters were set as follows: 10 ppm precursor and 0.6 Da product ion tolerances; static carbidomethylation (+ 57.021 Da) for cysteine; dynamic oxidation (+ 15.995 Da) for methionine; dynamic phosphorylation (+ 79.966 Da) for serine, threonine, and tyrosine. The Percolator peptide-spectrum matching validator located within the Proteome Discoverer software was used to validate PSMs. Proteins unable to be differentiated by MS/MS analysis alone because of similar peptide composition were grouped using the principles of parsimony. The report consisting of identities and spectrum counts (number of PSMs) for each protein was then exported to serve as a semi-quantitative measure of relative protein levels detected in each AH sample.

### OCT and HRT measurements

Retinal nerve fiber layer (RNFL) thickness was calculated using SPECTRALIS Tracking Laser Tomography (Heidelberg Engineering, Franklin, MA, USA). Via this system, a 24-line high resolution radial scan of the optic nerve head centered on the Bruch’s membrane opening (BMO) was acquired. The neuroretinal rim was subsequently defined as the region between the BMO and the nearest point on the internal limiting membrane based on this image. RNFL thickness was determined utilizing three circular scans centered on the optic nerve head delineated by the BMO. These findings were then compared to a reference database of healthy eyes adjusted for BMO size and age and presented in the Garway-Heath sector format, facilitating structural and functional correlations. Additionally, the ganglion cell layer (GCL) and the macula were comprehensively evaluated by the multi-layer segmentation software, the results of which were illustrated using a GCL thickness map.

Imaging of the optic nerve head was also performed using the Heidelberg Retinal Tomograph (Heidelberg Engineering, Franklin, MA, USA). A set of 3D images of the optic nerve and surrounding retina at various depths were collected and collated to create a 3D representation of the whole optic nerve. From this, a set of stereometric parameters (e.g., cup-to-disc area ratio, cup shape measure, and rim volume) were calculated. The cup-to-disc area ratio evaluates the extent of cupping relative to the area of the optic disc. In patients with uncontrolled glaucoma, the cup-to-disc area increases over time. The cup shape measure numerically encapsulates the configuration of the cup, incorporating an assessment of both the steepness of cup walls and variation in depth. In patients without evidence of glaucomatous damage, this metric is generally negative. Conversely, for glaucomatous eyes, this value is typically less negative or positive [16]. Finally, rim area assesses the neuroretinal rim, which forms the demarcation between the cup and disc. Among patients with glaucoma, this metric is typically lower, reflecting degradation or loss of the nerve fibers that compose the neuroretinal rim.

### Statistical analyses

All statistical analyses were performed using the R project for Statistical Computing (version 3.6.3). The two-tailed t-test and chi-square tests were utilized to compare the clinical characteristics of POAG and cataract patients. Prior to data analysis, PSM values derived from LC-MS/MS analysis were quantile normalized and the proportion of samples in which each protein was detected was determined. The negative binomial regression model was fitted to assess the disease-specific differences in apolipoproteins levels. Correlations between apolipoproteins and clinical parameters (e.g., OCT and HRT measures) were assessed through Spearman’s correlation coefficients. The threshold for significance was set at *p* < 0.05.

## Results

### Apolipoprotein levels in human aqueous humor

**Fig. 1:**
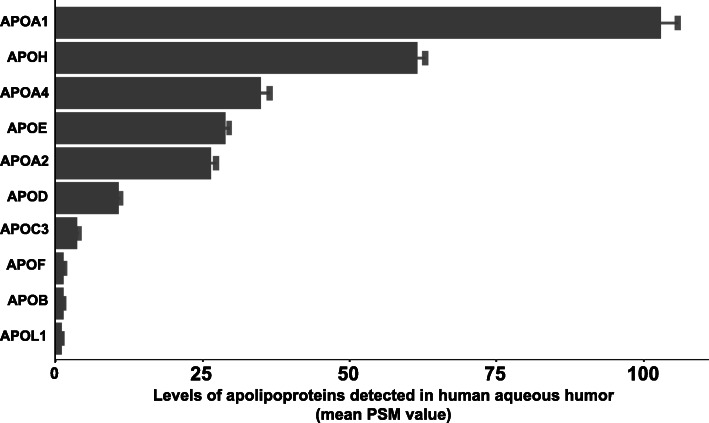
Levels of the ten apolipoproteins detected in 231 human aqueous humor samples

A total of 21 apolipoproteins are known to be a part of the human proteome [17], 10 of which we identified in the 231 AH samples analyzed (Fig. 1). The mean PSM values of all apolipoproteins and the proportion of samples in which they were detected are listed in Table 2. Three of the 10 apolipoproteins were detected in fewer than 10% of samples and were therefore excluded from further statistical analyses. Six of the other seven apolipoproteins were detected in 100% of samples, while the seventh was detected in 57% of our samples. These seven most abundant apolipoproteins were APOA1 (mean PSM ± SD, 102.88 ± 44.25), APOH (61.44 ± 21.30), APOA4 (34.88 ± 23.25), APOE (28.82 ± 11.41), APOA2 (26.36 ± 14.09), APOD (10.62 ± 5.10), and APOC3 (3.63 ± 2.80).

**Table 2:** Levels of apolipoproteins in human aqueous humor

Gene Symbol	Description	Mean PSM Value (± SD)	Detected in Proportion of Samples (%)
APOA1	Apolipoprotein A-I	102.88 ± 44.25	100
APOH	Beta-2-glycoprotein 1	61.44 ± 21.30	100
APOA4	Apolipoprotein A-IV	34.88 ± 23.25	100
APOE	Apolipoprotein E	28.82 ± 11.41	100
APOA2	Apolipoprotein A-II	26.36 ± 14.09	100
APOD	Apolipoprotein D	10.62 ± 5.10	100
APOC3	Apolipoprotein C-III	3.63 ± 2.80	57.14
APOF	Apolipoprotein F	1.29 ± 0.51	7.36
APOB	Apolipoprotein B-100	1.23 ± 0.53	8.23
APOL1	Apolipoprotein L1	1.05 ± 0.1	5.63
APOA5	Apolipoprotein A-V	ND	0
APOC1	Apolipoprotein C-I	ND	0
APOC2	Apolipoprotein C-II	ND	0
APOC4	Apolipoprotein C-IV	ND	0
APOL2	Apolipoprotein L-II	ND	0
APOL3	Apolipoprotein L-III	ND	0
APOL4	Apolipoprotein L-IV	ND	0
APOL5	Apolipoprotein L-V	ND	0
APOL6	Apolipoprotein L-VI	ND	0
APOM	Apolipoprotein M	ND	0
APOO	Apolipoprotein O	ND	0

*ND* Not Detected

### POAG-specific alterations in human aqueous humor apolipoproteins

**Fig. 2:**
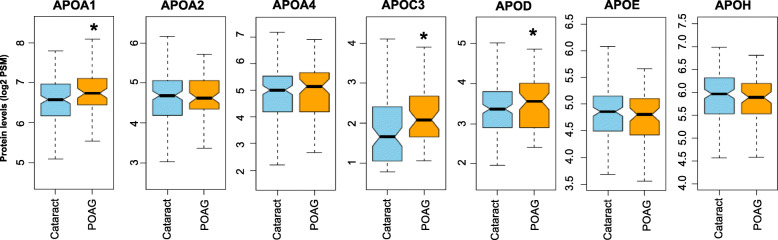
Levels of three apolipoproteins were significantly increased in subjects with primary open angle glaucoma (POAG) compared to subjects with cataracts alone, the controls (C). The boxplots depict the distribution of protein levels in each group. Peptide spectrum match (PSM) values were used as semi-quantitative measurement of protein levels. The raw PSM values were log2 transformed to achieve a normal distribution

Statistical analyses were performed to determine the POAG-specific alterations in aqueous humor apolipoproteins using all 231 samples (Fig. 2). Fold change (FC) values represent the change in protein levels in the POAG group (*n* = 158) compared to the cataract group (*n* = 73). The levels of three proteins, APOA1 (FC = 1.17; *p* = 0.01), APOC3 (FC = 1.25; *p* = 0.02), and APOD (FC = 1.13; *p* = 0.04), were significantly higher in POAG patients as compared to cataract patients (Table 3**)**.

**Table 3:** Race and sex specific changes in the levels of apolipoproteins in POAG subjects

		APOA1	APOA2	APOA4	APOC3	APOD	APOE	APOH
		(Mean PSM ± SD)	(Mean PSM ± SD)	(Mean PSM ± SD)	(Mean PSM ± SD)	(Mean PSM ± SD)	(Mean PSM ± SD)	(Mean PSM ± SD)
Overall	Cataract	97.29 ± 39.78	25.44 ± 12.16	33.94 ± 23.69	3.3 ± 2.79	10.09 ± 4.74	29.15 ± 10.79	62.39 ± 21.26
	POAG	114.97 ± 50.87*	28.34 ± 17.49	36.91 ± 22.31	4.21 ± 2.77*	11.75 ± 5.67*	28.08 ± 12.68	59.4 ± 21.4
Male	Cataract	111.95 ± 39.55	29.03 ± 14.01	42.26 ± 23.82	3.79 ± 2.78	10.14 ± 3.96	28.32 ± 9.69	67.47 ± 21.46
	POAG	107.84 ± 44.59	26.64 ± 11.7	38.47 ± 20.76	3.96 ± 2.49	10.9 ± 5.11	26.69 ± 10.23	58.3 ± 23.67
Female	Cataract	88.79 ± 37.56	23.36 ± 10.46	29.11 ± 22.34	2.97 ± 2.77	10.07 ± 5.16	29.64 ± 11.4	59.44 ± 20.68
	POAG	119.94 ± 54.78*	29.52 ± 20.65*	35.82 ± 23.5*	4.35 ± 2.96*	12.34 ± 6.01*	29.05 ± 14.18	60.16 ± 19.93
Caucasian	Cataract	113.2 ± 36.86	30.57 ± 11.98	42.15 ± 23.49	3.01 ± 2.3	10.16 ± 4.74	30.75 ± 11.32	71.39 ± 20.65
	POAG	112.58 ± 43.7	27.75 ± 11.8	39.24 ± 25.54	3.92 ± 2.15	10.63 ± 4.79	25.03 ± 9.09*	56.8 ± 24.06*
African American	Cataract	86.74 ± 38.27	22.04 ± 11.08	28.49 ± 22.32	3.44 ± 3.01	10.05 ± 4.77	28.09 ± 10.35	56.42 ± 19.58
	POAG	116.54 ± 55.52*	28.72 ± 20.52*	35.37 ± 20.06*	4.36 ± 3.08	12.49 ± 6.12*	30.1 ± 14.32	61.11 ± 19.56

**Fig. 3:**
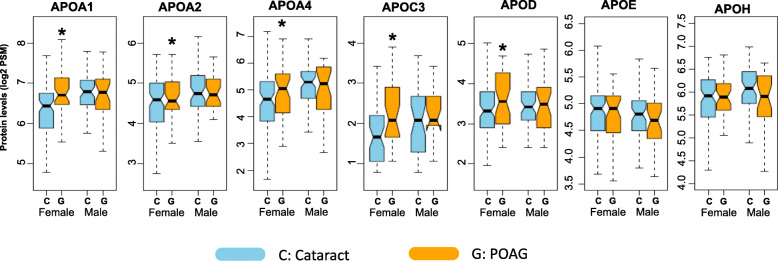
Levels of five apolipoproteins were significantly increased in female subjects with primary open angle glaucoma (G) compared to subjects with cataracts alone, the controls (C). No significant alterations in apolipoproteins were detected in male subjects. The boxplots depict the distribution of protein levels in each group

Demographic variation in these apolipoproteins was investigated by further stratification of our subjects according to gender and race. Within the male subset, no significant differences in apolipoprotein levels were found between POAG and cataract subjects. Conversely, female POAG patients had significantly greater levels of APOA1 (FC = 1.36; *p* < 0.01), APOA2 (FC = 1.21; *p* = 0.03), APOA4 (FC = 1.30; *p* = 0.04), APOC3 (FC = 1.38; *p* = 0.01), and APOD (FC = 1.20; *p* = 0.04) compared to cataract patients (Fig. 3).

**Fig. 4:**
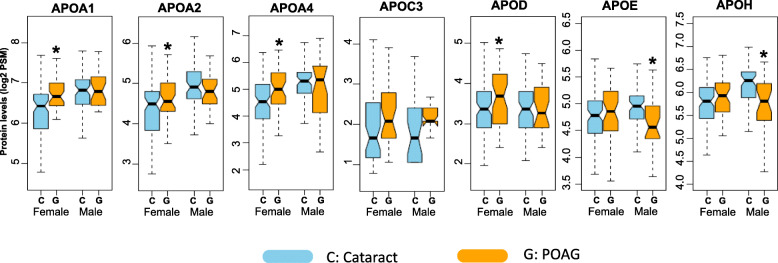
Levels of four apolipoproteins were significantly increased in African American subjects with primary open angle glaucoma (G) compared to subjects with cataracts alone, the controls (C). Levels of two apolipoproteins were significantly decreased in Caucasian subjects with primary open angle glaucoma compared to subjects with cataracts alone. The boxplots depict the distribution of protein levels in each group

When parsed along the lines of race, two apolipoprotein levels were found to be lower in the Caucasian POAG subset: APOE (FC = 0.82; *p* = 0.02) and APOH (FC = 0.76; *p* = 0.01). In the African American POAG subset, significant upregulation of four apolipoproteins was noted: APOA1 (FC = 1.34; *p* < 0.01), APOA2 (FC = 1.25; *p =* 0.02), APOA4 (FC = 1.37; *p* = 0.01), and APOD (FC = 1.19; *p =* 0.04) (Fig. 4).

### Correlation of human aqueous humor apolipoproteins with RNFL thickness

Two of the seven apolipoproteins were significantly correlated with RNFL thickness across the 231 subjects: APOC3 (ρ = − 0.25; *p* = 0.01) and APOD (ρ = − 0.31; *p* < 0.01. When these subjects were stratified by disease state, only the association between APOD levels and RNFL thickness remained significant in the POAG subset (ρ = − 0.44; *p <* 0.01).

### Correlation of human aqueous humor apolipoproteins with HRT parameters

The association of the seven apolipoproteins with HRT parameters are recorded in Table 4. APOC3 levels were positively correlated with cup shape measure (ρ = 0.37; *p* = 0.01), which persisted in the cataract subset (ρ = 0.39; *p* = 0.01). Contrastingly, amongst the POAG subgroup, levels of APOA1 (ρ = − 0.51; *p* < 0.01), APOA2 (ρ = − 0.49; *p* < 0.01), and APOH (ρ = − 0.61; *p* < 0.01) had a negative correlation with cup shape measure.

**Table 4:** Apolipoproteins significantly correlated with clinical parameters

Proteins	Clinical Variables	Overall		Cataract		POAG	
		Corr.	*P*-value	Corr.	*P-*value	Corr.	*P*-value
*OCT Parameters*							
APOC3	RNFL avg. thickness	−0.25	0.01*	−0.25	0.06	−0.22	0.20
APOD	RNFL avg. thickness	−0.31	< 0.01*	0.00	0.99	−0.44	< 0.01*
*HRT Parameters*							
APOA1	Cup Shape Measure	0.00	0.98	0.19	0.09	− 0.51	< 0.01*
APOA2	Cup Shape Measure	−0.03	0.74	0.15	0.18	−0.49	0.01*
APOC3	Cup Shape Measure	0.37	0.01*	0.39	0.01*	0.39	0.17
APOH	Cup Shape Measure	−0.07	0.45	0.18	0.10	−0.61	< 0.01*
APOC3	Rim Volume (mm^2^)	−0.29	0.02*	−0.25	0.12	−0.27	0.24
APOE	Rim Volume (mm^2^)	0.27	< 0.01*	0.36	< 0.01*	0.17	0.31
APOE	Cup to Disc Area ratio	−0.19	0.04*	−0.32	< 0.01*	− 0.08	0.63
APOH	Cup to Disc Area ratio	−0.21	0.02*	−0.03	0.77	−0.37	0.02*

*Corr.* Correlation coefficient

The cup-to-disc ratio was negatively associated with APOE (ρ = − 0.32; *p* < 0.01) and APOH (ρ = − 0.21; *p* = 0.02) in the overall sample set. This negative correlation persisted for APOE in the cataract subset (ρ = − 0.32; *p* < 0.01) and APOH in the POAG subset (ρ = − 0.37; *p* = 0.02).

Rim volume was negatively associated with APOC3 level (ρ = − 0.29; *p* = 0.02), but positively associated with APOE (ρ = 0.27; *p* < 0.01). Stratification by POAG and cataract status only retained the positive correlation between APOE and rim volume in the cataract subset (ρ = 0.36; *p* < 0.01).

## Discussion

Examination of variations in the AH proteome between disease states as well as between demographic groups may prove helpful in understanding the elusive mechanism(s) of glaucomatous optic neuropathy. Apolipoproteins have been previously established as markers for multiple systemic [18–20] and neurodegenerative diseases [21–25], and it has been more recently observed that their levels in AH are altered in the presence of ocular conditions [8, 10]. As such, we aimed to probe our large data set for differences in AH apolipoprotein levels between glaucomatous and cataract patients and quantify intra-population variation. In both of these areas, we discovered interactive effects that suggest disease-specific alterations in apolipoprotein levels vary between demographic groups.

We detected 10 apolipoproteins in our AH samples. After excluding the three low-incidence apolipoproteins (APOB, APOF, APOL1), the remaining seven (APOA1, APOA2, APOA4, APOC3, APOD, APOE, and APOH) were abundant in the AH proteome. The detection of APOA1, APOA2, APOA4, APOD, APOE, and APOH is consistent with the findings of other studies [3, 12, 26].

When considering all 231 subjects, APOA1, APOC3, and APOD were significantly higher among patients with POAG. This agrees with previous investigations wherein elevated levels of both APOA1 and APOC3 were detected among individuals with glaucoma [12, 27]. APOA1 levels, in particular, have demonstrated potential value for the detection of primary chronic angle-closure glaucoma as the component of a proposed diagnostic protein panel [28]. Leveraging such a finding to therapeutic effect remains difficult as mechanistic linkages to the optic neuropathy remain to be discovered. Investigations of other ocular conditions (e.g., diabetic retinopathy, age-related maculopathy) have posited that increased APOA1 levels may indicate underlying inflammatory processes [29, 30]. Although there is a robust corpus of literature that implicates neuro-inflammation in glaucomatous optic neuropathy [31], further research is needed to elucidate pathophysiologic connections.

Overall, only three apolipoproteins were significantly higher among glaucomatous patients. However, these findings were different in male and female subgroups, suggesting an interactive effect between disease state, gender, and apolipoprotein levels. No apolipoprotein levels were found to be altered in males based on disease state, while five apolipoprotein (APOA1, APOA2, APOA4, APOC3, and APOD) levels were increased in females with POAG. Several studies have previously detected significant gender-specific differences in systemic (serum) apolipoprotein levels [13, 14, 32]. Schaefer *et. al.* observed an elevated plasma level of APOA1 among females (124 ± 24 mg/dl) relative to males (108 ± 16 mg/dl) [13]. A more recent investigation by Anagnostis *et. al.* reported similar findings and further identified an interaction between menopause status, age, and plasma concentrations of APOA1, APOA2, and APOB in females [14]. However, heretofore, limited research has extended into human AH. Perumal *et. al.* reported gender-dependent changes in the AH proteome [15]. The present investigation extends such findings by suggesting gender-specific differences in POAG. Further research is required to delineate whether such findings distill along structural, endocrine or other lines. Support for the structural vantage point comes from reports indicating females as having average shorter axial lengths and shallower anterior chamber depths [33–36]. Support for the endocrine aspect comes from reports proposing a protective effect of endogenous estrogen for retinal ganglion cells in pre-menopausal females [37]. The authors suggested mechanisms relating to extracellular matrix production and cellular signaling [38–40]. Regardless, further characterization of pathophysiologic differences between the genders and POAG is necessary to explain the variance in apolipoprotein levels.

We also observed interactions between race and AH apolipoprotein levels. Within the African American cohort, APOA1, APOA2, APOA4, and APOD levels were significantly higher among glaucoma patients. Alternatively, the Caucasian glaucomatous cohort had lower levels of APOE and APOH. These findings pose multiple questions. First, does this divergence explain on some level the increased incidence, progression, and severity of glaucoma in the African American population [41, 42]? Second, does race predict different disease patterns and treatment responses? If so, analyses with specific racial compositions of patients may be necessary for personalized treatment algorithms. Third, while prior investigations have reported increased levels of APOE among glaucomatous subjects [12, 43], our study did not confirm this. Instead, we found APOE levels decreased among glaucomatous patients only within the Caucasian subset. Accordingly, it is important for future studies characterizing the AH proteome to further investigate the impact of race on the AH proteome when interpreting findings to reduce this as a potential confounding factor.

Assessment of the relationship between OCT parameters and AH apolipoprotein levels demonstrated a negative correlation between average RNFL thickness and two apolipoproteins (APOC3 and APOD). However, this association only persisted with APOD amongst the glaucoma subset. Interestingly, this is the same apolipoprotein which has emerged as a potential candidate for evaluation in pseudoexfoliative glaucoma and the neurodegenerative conditions of multiple sclerosis and Alzheimer’s disease [44–48]. Among glaucomatous patients, APOD has previously been demonstrated to correlate with functional visual field parameters including Pattern Standard Deviation (PSD), Visual Field Index (VFI), Mean Deviation (MD), and Glaucoma Hemifield Test (GHT) [49]. Lipids and cholesterol are conserved following neurodegenerative processes [50, 51], and therefore elevated APOD levels may reflect increased recycling of these substances during regeneration. This, in turn, may promote neuritogenesis and synaptogenesis in these patients [52]. Taken together, these results suggest that higher levels of APOD may facilitate increased regeneration in patients with glaucomatous damage.

Previously, APOC3 has been implicated in certain ocular conditions. Increased APOC3 levels have been observed in patients with branch retinal vein occlusion with macular edema. It is proposed as interrelating with inflammatory processes [53]. Other studies have also elucidated a linkage between POAG and APOC3 [10, 27, 54]. In these studies, APOC3 levels were also upregulated. Although APOH was associated with HRT parameters in POAG patients, there remains a limited understanding of its role within the neurodegenerative condition. Despite its implication in other ocular diseases such as proliferative diabetic retinopathy [55, 56], further characterization of the role of apolipoproteins in the process of glaucoma has yet to be conducted.

Despite an abundance of literature grossly analyzing differences in the AH between patients with and without POAG [3, 10, 12, 27], to our knowledge, studies evaluating the influence of demographics are limited. In addition, studies analyzing gender and race-specific differences in the AH proteome did not examine different disease states [2, 15]. Therefore, our study extends knowledge to identify how the interaction between demographic characteristics and POAG affects apolipoprotein levels in human AH.

Our study possesses multiple strengths, including a large sample set and utilization of one of the latest LC-MS/MS technologiesy for identification of proteins from small volume samples [57, 58]. One of the limitations of this study is lack of quantification of the lipid content in human AH. Other investigations have reported that the AH lipid profiles of patients with POAG are distinct from controls. Those studies have suggested the differential regulation of lipids such as 12,13-DiHOME and platelet activating factor, among others, is implicated in inflammatory processes [59, 60]. Because lipids may be an essential component of the oxidative stress-related pathogenesis of POAG, further studies into the relationship between AH lipid content and apolipoprotein levels are required. Another limitation is our utilization of cataract patients as the control group. While these individuals have been traditionally employed in studies involving the AH [10–12, 27], cataract formation is associated with alterations in multiple proteins, including β-crystallin and keratin proteins [26, 54]. Accurate quantification of differences associated with disease states such as POAG would require the collection of control samples from healthy adults, an ethically unfeasible task.

## Conclusions

In the present study, through utilization of a large sample set, we identified six apolipoproteins detected in all AH samples and one apolipoprotein detected in 57% of samples. Analysis of the relationships between these proteins and disease state, patient demographics, and clinical parameters of glaucomatous damage demonstrated multiple interactive effects. These differential associations emphasize the necessity of considering demographic factors including race and gender when analyzing a heterogeneous population. The elucidation of whether apolipoprotein alterations represent causative, degenerative, and/or reparative processes is important and warrants further study. Leveraging such results to treat glaucoma, detect its presence, or assess adequacy of treatment would be a boon to human vision.

## Supplementary Information

**Additional file 1.**

## Data Availability

The datasets used in the current study are available as Supplemental Table-1.
